# Editorial: Microbiome and machine learning, volume II

**DOI:** 10.3389/fmicb.2024.1499260

**Published:** 2024-10-15

**Authors:** Domenica D'Elia, Aldert Zomer, Isabel Moreno Indias, Erik Bongcam-Rudloff, Randi Jacobsen Bertelsen, Marcus Joakim Claesson

**Affiliations:** ^1^Department of Biomedical Sciences, National Research Council, Institute for Biomedical Technologies, Bari, Italy; ^2^Department of Biomolecular Health Sciences (Infectious Diseases and Immunology), Faculty of Veterinary Medicine, Utrecht University, Utrecht, Netherlands; ^3^Department of Endocrinology and Nutrition, Virgen de la Victoria University Hospital, The Biomedical Research Institute of Malaga and Platform in Nanomedicine (IBIMA-BIONAND Platform), University of Malaga, Malaga, Spain; ^4^Swedish University of Agricultural Sciences, Department of Animal Breeding and Genetics, Uppsala, Sweden; ^5^Department of Clinical Science, Faculty of Medicine, University of Bergen, Bergen, Norway; ^6^School of Microbiology and APC Microbiome Ireland, University College Cork, Cork, Ireland

**Keywords:** microbiome, machine learning, explainable artificial intelligence, standards, best practices

Microbiomes play a crucial role in various biological processes, ranging from human and animal health to the functioning soil and marine ecosystems that support food production and biodiversity. Understanding how perturbations of these communities can impact their respective environments is essential for making new scientific discoveries and developing practical solutions to improve both human wellbeing and the health of our planet. However, encapsulating the sheer diversity of microbial communities and the intricate web of interactions they establish with other organisms results in vast and complex datasets. Traditional statistical methods often fall short in capturing both the nuances and global summary of these interactions. With its ability to process large datasets and identify intricate patterns, machine learning (ML) provides a powerful solution. Techniques such as neural networks and ensemble learning models are particularly well-suited for this task, enabling researchers to make sense of the multi-layered structures inherent in microbiome data. Nevertheless, the integration of ML in microbiome research has challenges, including input data standardization, heterogenous, noisy, and high-dimensional data as well as interpretability of ML models. Addressing these challenges requires a concerted effort from biologists, data scientists, and computational experts, fostering a collaborative environment where knowledge and techniques can be shared and refined. This is a exactly what we carried out as part of the COST Action ML4Microbiome (CA18131), which is best summarize by publications in the “Microbiome and Machine Learning” volumes in Frontiers in Microbiology. This second volume represents a significant step forward in harnessing the power of artificial intelligence to decode the complex world of microbiomes.

ML4Microbiome key achievements are summarized in D'Elia et al.. In this article, the authors also underscore the importance of ethical considerations when deploying machine learning in microbiome research. Ensuring data privacy, avoiding biases in algorithmic predictions, and promoting transparency in model development are essential to maintaining public trust and maximizing the societal benefits of these technologies. Papoutsoglou et al. subsequently detailed the technical complexity of applying ML for microbiome research. The review identifies and addresses challenges such as preprocessing, feature selection, predictive modeling, performance estimation, and model interpretation, finally providing a set of recommendations on algorithm selection, pipeline creation, and evaluation to aid in decision-making processes related to microbiome research. An in-depth exploration of data preprocessing methods is provided by Ibrahimi et al.. This article aims to guide both established researchers and those new to the field in selecting appropriate transformation methods based on their research questions, objectives, and data characteristics.

To provide researchers with insights into specific ML resources facilitating microbiome analysis, Marcos-Zambrano et al. categorized ML tools based on the type of analysis they are designed for and the ML algorithms they employ. The focus spans various software tools for feature generation, taxonomic assignment, clustering, binning, and disease classification.

Kumar et al. emphasize the crucial role of metadata in interpreting and comparing microbiome datasets and highlight the need for standardized metadata protocols to fully leverage the potential of metagenomic data. In this paper microbiome data are classified into five types based on the methodology used for their production: shotgun sequencing, amplicon sequencing, metatranscriptomic sequencing, metabolomic measurements, and metaproteomic expression analysis. The significance of metadata in data interpretation and comparison and the challenges in collecting standardized metadata are thoroughly explored.

In the clinical domain, Chang et al. investigated the diagnostic classification and predictive power of four different ML methods for diagnostic screening in myasthenia gravis (MG) using gut microbiome data. The proposed ML model may serve as biomarkers for clinical use and can be applied for non-invasive screening of MG. Zhang et al. present a study that provides valuable insights into the potential impact of gut microbiota on carcinoid syndrome (CS). The article investigates the cause-and-effect relationship between gut microbiota abundance and carcinoid syndrome (CS) through a bidirectional Mendelian randomization study. Murovec et al. present a study aimed to compare microbiome profiles of patients with colorectal cancer (CRC) and colorectal adenomas (CRA) to healthy participants using metagenomic data. The methodology involved extensive analysis using the MetaBakery pipeline, integrating data matrices like microbial taxonomy, functional genes, enzymatic reactions, metabolic pathways, and predicted metabolites. By integrating all layers of information, the study showcased the development of robust prediagnostic methods for colorectal cancer detection.

To analyze microbiome data in the context of identifying biomarkers for colorectal cancer (CRC) Novielli et al. centered their study on leveraging explainable artificial intelligence (XAI). By employing ML techniques, the researchers aimed to classify a cohort of control subjects from those with CRC based on gut microbiota data and demographic information. The study underscored the potential of gut microbiota data within an XAI framework for precise CRC classification. Another study underscoring the importance of combining ML and XAI approaches is presented by Magarelli et al.. In this study, the researchers explored the use of ML algorithms, specifically the Random Forest (RF) classifier, to effectively classify the geographical origin of PDO Mozzarella di Bufala Campana based on microbiota data. The results showed that the RF classifier outperformed other algorithms, achieving high accuracy in discerning the origin of the samples. The study emphasized the critical role of microbiota analysis in ensuring the authenticity, quality, and safety of food products. Another innovative approach of using XAI is presented by Tangaro et al.. This article outlines a comprehensive study protocol for understanding the interplay among human microbiota, volatilome, and disease biomarkers in Behçet's disease (BD). The study design involves a three-phase approach, including a clinical study with control and experimental groups receiving a soluble fiber-based dietary supplement alongside standard therapy, followed by data collection and analysis using gas chromatography, mass spectrometry, and metagenetic analysis to examine microbiota and volatilome composition. The third phase introduces XAI to analyze collected data to identify markers associated with BD, dietary habits and the dietary supplement, aiming to establish correlations between microbiota, volatilome, and phenotypic characteristics. The results demonstrate how the use of XAI algorithms on multi-modal clinical data could revolutionize disease management.

The importance of practical applications of ML in industries, particularly in the fields of probiotics and pharmaceuticals is exemplified in the article by Liu et al., who were able to discriminate between *Bifidobacterium longum* subsp. infantis and subsp. longum by leveraging MALDI-TOF MS and ML techniques. Through the application of logistic regression, RF, and support vector machine, the researchers developed classification models to differentiate between the two subspecies. The RF model emerged as the most effective. Overall, this study underscores the potential of combining MALDI-TOF MS and ML for rapid and precise discrimination of *Bifidobacterium* subspecies essential for product development and quality control, paving the way for microbial identification and classification advancements.

While these comparative method evaluations are indisputably important, the development of new tools for analyzing microbiome data is also pivotal for aiding the rapidly evolving field of microbiome research. Bakir-Gungor et al. present microBiomeGSM that can identify taxonomic biomarkers from metagenomic data using a new grouping, scoring and modeling (GSM) approach. The tool incorporates pre-existing taxonomy information into a ML model to analyze metagenomic datasets associated with different diseases. By focusing on specific taxonomic levels (genus, family, and order), microBiomeGSM aims to identify their associations with diseases and facilitate disease diagnosis.

Another article by Ligeti et al. introduces the ProkBERT model family, a series of genomic language models developed for microbiome applications. By utilizing the novel Local Context-Aware tokenization technique, the ProkBERT models exhibit superior performance in various tasks such as promoter prediction and phage identification for both supervised and unsupervised tasks. Importantly, the study emphasizes the significance of innovative approaches in leveraging the vast repositories of raw sequence data and navigating the complexities of labeling inconsistencies within the microbiology field.

Murovec et al. finally presents the development and utilization of MetaBakery, an integrated application designed as a framework for executing the bioBakery workflow on metagenomic sequencing data. MetaBakery streamlines the processing of paired or unpaired fastq files, with optional compression, using programs such as KneadData, MetaPhlAn, HUMAnN, and StrainPhlAn, along with integrated utilities. It includes MelonnPan for metabolite prediction and Mothur for calculating microbial alpha diversity. The development and utilization of MetaBakery provide a versatile and well-documented tool for microbiome analysis, offering efficient exploration of changing parameters and input datasets for various biostatistical and ML approaches.

In conclusion, as we continue to push the boundaries of what is possible at the intersection of microbiome science and ML, the potential applications are vast and varied. By bridging these two dynamic fields, we are paving the way for groundbreaking discoveries that have the potential to revolutionize science and improve lives. From enhancing our understanding of microbial ecology to developing novel diagnostic tools and treatments, the research showcased in this volume is a testament to the innovative and interdisciplinary nature of this field.

